# 4-Benzyl­pyridinium hydrogen selenate

**DOI:** 10.1107/S1600536808033801

**Published:** 2008-10-22

**Authors:** Wassim Maalej, Zakaria Elaoud, Tahar Mhiri, Abdelaziz Daoud, Ahmed Driss

**Affiliations:** a Laboratoire de l’Etat Solide, Faculté des Sciences de Sfax, BP 1171, 3000 Sfax, Tunisia; b Laboratoire de Matériaux et Cristallochimie, Département de Chimie, Faculté des Sciences, 2092 Campus Universitaire, Tunis, Tunisia

## Abstract

The structure of the title salt, C_12_H_12_N^+^·HSeO_4_^−^, consists of infinite parallel two-dimensional planes built of 4-benzyl­pyridinium and hydrogen selenate ions that are mutually connected by strong O—H⋯O and N—H⋯O hydrogen bonds. There are no contacts other than normal van der Waals inter­actions between the layers.

## Related literature

For general background, see Fleck (2006 ▶); Baran *et al.* (2000 ▶). For related compounds, see: Ben Hamada & Jouini (2006 ▶); Kaabi *et al.* (2004 ▶); Ben Djemaa *et al.* (2007 ▶); Gowda *et al.* (2007 ▶). 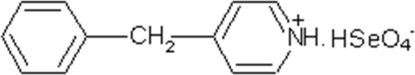


## Experimental

### 

#### Crystal data

C_12_H_12_N^+^·HSeO_4_^−^
*M* *_r_* = 314.19
Orthorhombic, 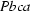

*a* = 27.449 (5) Å
*b* = 10.821 (6) Å
*c* = 8.830 (1) Å
*V* = 2623 (2) Å^3^
*Z* = 8
Mo *K*α radiation
μ = 2.87 mm^−1^
*T* = 289 (2) K
0.11 × 0.09 × 0.04 mm

#### Data collection

Enraf–Nonius CAD-4 diffractometer
Absorption correction: ψ scan (North *et al.*, 1968 ▶) *T*_min_ = 0.743, *T*_max_ = 0.894
3179 measured reflections
2817 independent reflections
1486 reflections with *I* > 2σ(*I*)
*R*_int_ = 0.045
2 standard reflections frequency: 120 min intensity decay: 11%

#### Refinement

*R*[*F* ^2^ > 2σ(*F* ^2^)] = 0.037
*wR*(*F* ^2^) = 0.087
*S* = 0.98
2817 reflections
207 parameters
H atoms treated by a mixture of independent and constrained refinement
Δρ_max_ = 0.28 e Å^−3^
Δρ_min_ = −0.31 e Å^−3^

### 

Data collection: *CAD-4 EXPRESS* (Enraf–Nonius, 1994 ▶); cell refinement: *CAD-4 EXPRESS*; data reduction: *XCAD4* (Harms & Wocadlo, 1995 ▶); program(s) used to solve structure: *SHELXS97* (Sheldrick, 2008 ▶); program(s) used to refine structure: *SHELXL97* (Sheldrick, 2008 ▶); molecular graphics: *ORTEPIII* (Burnett & Johnson, 1996 ▶) and *DIAMOND* (Brandenburg, 1998 ▶); software used to prepare material for publication: *WinGX* (Farrugia, 1999 ▶).

## Supplementary Material

Crystal structure: contains datablocks I, global. DOI: 10.1107/S1600536808033801/im2065sup1.cif

Structure factors: contains datablocks I. DOI: 10.1107/S1600536808033801/im2065Isup2.hkl

Additional supplementary materials:  crystallographic information; 3D view; checkCIF report

## Figures and Tables

**Table 1:** Hydrogen-bond geometry (Å, °)

*D*—H⋯*A*	*D*—H	H⋯*A*	*D*⋯*A*	*D*—H⋯*A*
O3—HO3⋯O4^i^	0.82	1.89	2.617 (4)	148
N—HN⋯O2^ii^	0.95 (4)	1.82 (5)	2.762 (5)	168 (4)

Symmetry codes: (i) 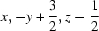; (ii) 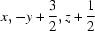.

**Fig. 1.**
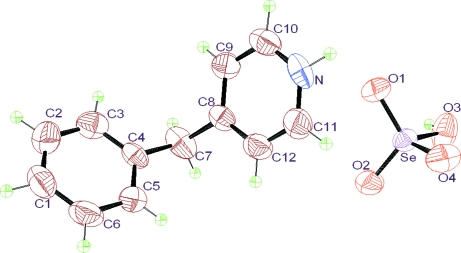
ORTEP drawing of [C6H5CH2C5H4NH]+ [HSeO4]-.

**Fig. 2.**
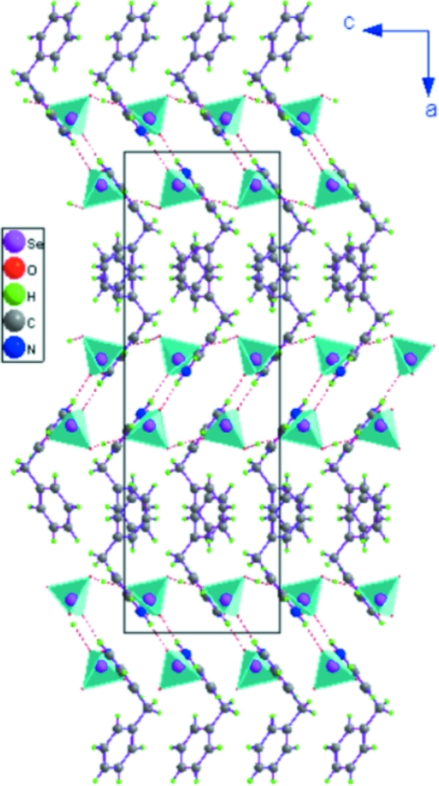
Projection along the b axis of the crystal structure of [C6H5CH2C5H4NH]+ [HSeO4]-.

## supplementary crystallographic information

### Comment

The title compound C_6_H_5_CH_2_C_5_H_4_NH^+^ HSeO_4_^-^ crystallizes in the orthorhombic space group Pbca. Correspondingly, there are eight formula units per unit cell. The crystal is built up of monohydrogenselenate anions connected by hydrogen bonds of the O—H···O type forming infinite chains [HSeO_4_]_n_^n-^ perpendicular to the [001] directions.

These chains are themselves interconnected by means of N—H···O hydrogen bonds originating from the [C_6_H_5_CH_2_C_5_H_4_NH]^+^ cation, so as to build a three-dimensional network. The distances Se—O in the [HSeO_4_]^-^ anions range from 1.608 (3) to 1.705 (3) Å (Fig. 1). These values are comparable to reported data (Fleck, 2006). The longest Se—O(2) distance of 1.705 (3) Å, is due to the presence of the acidic hydrogen atom on the SeO_4_ tetrahedron (Baran *et al.*, 2000).

The organic groups are located in the (011) planes at *x* = 1/4 and *x* = 3/4. The average values of the C—C, C—N and C═C bond lengths of 1.5165 (6) Å, 1.3305 (6) Å and 1.3753 (6) Å in the [C_6_H_5_CH_2_C_5_H_4_NH]^+^ cation are similar to those observed in related compounds (Ben Hamada & Jouini, 2006; Kaabi *et al.*, 2004; Ben Djemaa *et al.*, 2007; Gowda *et al.*, 2007). The C—C perpendicular interplanar distances range from 1.74 to 4.83 Å and the dihedral angle between two planes of rings is 67.04°, indicating the existence of strong van der Waals interactions by contacts between [C_6_H_5_CH_2_C_5_H_4_NH]^+^ cations.

All the hydrogen bonds (D—H···O, Table 1) and the van der Waals contacts give rise to a three dimensional network in the crystal structure.

### Experimental

Crystals of the title compound C_6_H_5_CH_2_C_5_H_4_NH^+^ HSeO_4_^-^ were prepared by slowly adding, at room temperature, an equimolecular proportion of H_2_SeO_4_ (1.9 cm^3^) to a solution of 4-benzyl pyridine (5 cm^3^). A crystalline precipitate was formed. After dissolving the precipitate by adding H_2_O, the solution is allowed to slowly evaporate at room temperature for several days until the formation of pink prismatic crystals with dimensions suitable for a crystallographic study occurs.

### Refinement

Hydrogen atoms at C_6_, C_9_ and O_3_ were positioned geometrically, with C—H = 0.93 Å and O—H = 0.82 Å, and were refined with *U*_iso_(H) = 1.41*U*_eq_ of the corresponding parent atom. The other H atoms bound to C (cyclic and CH_2_) groups, and N atoms were located from the difference Fourier map, and refined with distance restraints of [C—H = 0.90 (4)–1.00 (5) Å and *U*_iso_(H) = 0.06 (1)–0.10 (2) Å^2^, (cyclic groups)], [C—H = 0.88 (6)–1.00 (4) Å and *U*_iso_(H) = 0.06 (1)–0.12 (2) Å^2^, (CH_2_ group)], and N—H = 0.95 (4) Å with *U*_iso_(H) = 1.14*U*_eq_(N) for NH bond.

### Crystal data

**Table:**

C_12_H_12_N^+^·HSeO_4_^−^	*F*(000) = 1264
*M**_r_* = 314.19	*D*_x_ = 1.592 Mg m^−^^3^
Orthorhombic, *P**b**c**a*	Mo *K*α radiation, λ = 0.71073 Å
Hall symbol: -P 2ac 2ab	Cell parameters from 25 reflections
*a* = 27.449 (5) Å	θ = 14–16°
*b* = 10.821 (6) Å	µ = 2.87 mm^−^^1^
*c* = 8.830 (1) Å	*T* = 289 K
*V* = 2623 (2) Å^3^	Prism, pink
*Z* = 8	0.11 × 0.09 × 0.04 mm

### Data collection

**Table:**

Enraf–Nonius CAD-4 diffractometer	1486 reflections with *I* > 2σ(*I*)
Radiation source: fine-focus sealed tube	*R*_int_ = 0.045
graphite	θ_max_ = 27.0°, θ_min_ = 2.4°
Non–profiled ω/2θ scans	*h* = 0→35
Absorption correction: ψ scan (North *et al.*, 1968)	*k* = 0→13
*T*_min_ = 0.743, *T*_max_ = 0.894	*l* = −2→11
3179 measured reflections	2 standard reflections every 120 min
2817 independent reflections	intensity decay: 11%

### Refinement

**Table:**

Refinement on *F*^2^	H atoms treated by a mixture of independent and constrained refinement
Least-squares matrix: full	*w* = 1/[σ^2^(*F*_o_^2^) + (0.0299*P*)^2^] where *P* = (*F*_o_^2^ + 2*F*_c_^2^)/3
*R*[*F*^2^ > 2σ(*F*^2^)] = 0.037	(Δ/σ)_max_ = 0.001
*wR*(*F*^2^) = 0.087	Δρ_max_ = 0.28 e Å^−^^3^
*S* = 0.98	Δρ_min_ = −0.30 e Å^−^^3^
2817 reflections	Extinction correction: SHELXL97 (Sheldrick, 2008), Fc^*^=kFc[1+0.001xFc^2^λ^3^/sin(2θ)]^-1/4^
207 parameters	Extinction coefficient: 0.0065 (3)
0 restraints	

### Special details

**Table:**

Geometry. All e.s.d.'s (except the e.s.d. in the dihedral angle between two l.s. planes) are estimated using the full covariance matrix. The cell e.s.d.'s are taken into account individually in the estimation of e.s.d.'s in distances, angles and torsion angles; correlations between e.s.d.'s in cell parameters are only used when they are defined by crystal symmetry. An approximate (isotropic) treatment of cell e.s.d.'s is used for estimating e.s.d.'s involving l.s. planes.
Refinement. Refinement of *F*^2^ against ALL reflections. The weighted *R*-factor *wR* and goodness of fit *S* are based on *F*^2^, conventional *R*-factors *R* are based on *F*, with *F* set to zero for negative *F*^2^. The threshold expression of *F*^2^ > σ(*F*^2^) is used only for calculating *R*-factors(gt) *etc*. and is not relevant to the choice of reflections for refinement. *R*-factors based on *F*^2^ are statistically about twice as large as those based on *F*, and *R*- factors based on ALL data will be even larger.

### Fractional atomic coordinates and isotropic or equivalent isotropic displacement parameters (Å^2^)

**Table:**

	*x*	*y*	*z*	*U*_iso_*/*U*_eq_	
Se	0.568598 (14)	0.68653 (3)	0.34450 (4)	0.04502 (15)	
O1	0.53870 (10)	0.8139 (2)	0.3574 (4)	0.0652 (8)	
O2	0.53625 (10)	0.5754 (2)	0.2732 (4)	0.0685 (9)	
O3	0.61665 (10)	0.7080 (3)	0.2250 (4)	0.0779 (10)	
HO3	0.6065	0.7281	0.1411	0.11 (2)*	
O4	0.59571 (11)	0.6441 (3)	0.4996 (3)	0.0644 (8)	
C1	0.22067 (18)	0.5917 (5)	0.6315 (6)	0.0675 (14)	
C2	0.21967 (19)	0.6932 (5)	0.5412 (7)	0.0746 (15)	
C3	0.25920 (19)	0.7237 (5)	0.4540 (6)	0.0624 (13)	
C4	0.30049 (14)	0.6492 (4)	0.4530 (4)	0.0460 (10)	
C5	0.30108 (17)	0.5471 (4)	0.5468 (6)	0.0564 (12)	
C6	0.26129 (17)	0.5184 (4)	0.6359 (6)	0.0681 (14)	
HC6	0.2621	0.4494	0.6988	0.061 (13)*	
C7	0.3437 (2)	0.6798 (6)	0.3529 (6)	0.0683 (14)	
C8	0.38461 (14)	0.7417 (4)	0.4400 (4)	0.0462 (10)	
C9	0.38853 (17)	0.8693 (4)	0.4482 (5)	0.0538 (11)	
HC9	0.3662	0.9193	0.3977	0.046 (11)*	
C10	0.42554 (19)	0.9215 (5)	0.5313 (6)	0.0630 (13)	
C11	0.45454 (18)	0.7264 (5)	0.5991 (6)	0.0631 (13)	
C12	0.41870 (17)	0.6719 (5)	0.5168 (6)	0.0580 (12)	
N	0.45683 (14)	0.8484 (4)	0.6053 (5)	0.0621 (11)	
HC1	0.1926 (15)	0.573 (4)	0.690 (5)	0.077 (15)*	
HC2	0.1914 (18)	0.742 (5)	0.540 (6)	0.102 (18)*	
HC3	0.2621 (16)	0.795 (4)	0.388 (5)	0.082 (16)*	
HC5	0.3276 (13)	0.498 (4)	0.547 (4)	0.058 (13)*	
H1C7	0.334 (2)	0.729 (6)	0.280 (7)	0.12 (2)*	
H2C7	0.3573 (13)	0.602 (4)	0.309 (4)	0.062 (14)*	
HC10	0.4295 (16)	1.006 (4)	0.543 (5)	0.090 (17)*	
HC11	0.4786 (18)	0.680 (4)	0.661 (6)	0.104 (19)*	
HC12	0.4167 (16)	0.587 (5)	0.503 (5)	0.087 (16)*	
HN	0.4820 (15)	0.885 (4)	0.664 (5)	0.071 (14)*	

### Atomic displacement parameters (Å^2^)

**Table:**

	*U*^11^	*U*^22^	*U*^33^	*U*^12^	*U*^13^	*U*^23^
Se	0.0483 (2)	0.0418 (2)	0.0450 (2)	0.00559 (19)	0.0005 (2)	−0.0042 (2)
O1	0.0665 (18)	0.0466 (16)	0.082 (2)	0.0135 (15)	−0.0004 (18)	−0.0093 (17)
O2	0.0725 (19)	0.0479 (17)	0.085 (2)	0.0001 (16)	−0.0203 (18)	−0.0108 (17)
O3	0.0505 (17)	0.120 (3)	0.063 (2)	0.0120 (19)	0.0084 (16)	0.027 (2)
O4	0.093 (2)	0.0568 (17)	0.0429 (16)	0.0089 (17)	−0.0115 (16)	−0.0017 (15)
C1	0.057 (3)	0.070 (3)	0.075 (4)	−0.022 (3)	0.012 (3)	−0.016 (3)
C2	0.054 (3)	0.075 (4)	0.095 (4)	0.005 (3)	−0.007 (3)	−0.004 (4)
C3	0.075 (3)	0.055 (3)	0.057 (3)	−0.002 (3)	−0.013 (3)	0.008 (2)
C4	0.053 (3)	0.048 (2)	0.037 (2)	−0.019 (2)	0.001 (2)	−0.0074 (19)
C5	0.055 (3)	0.046 (3)	0.068 (3)	−0.001 (2)	0.007 (3)	0.001 (2)
C6	0.084 (4)	0.045 (3)	0.076 (4)	−0.013 (2)	0.016 (3)	0.013 (3)
C7	0.077 (3)	0.081 (4)	0.047 (3)	−0.028 (3)	0.010 (3)	−0.014 (3)
C8	0.053 (2)	0.050 (2)	0.036 (2)	−0.012 (2)	0.011 (2)	0.002 (2)
C9	0.060 (3)	0.051 (2)	0.050 (3)	−0.001 (2)	0.002 (2)	0.014 (2)
C10	0.072 (3)	0.051 (3)	0.066 (3)	−0.016 (3)	0.014 (3)	−0.006 (3)
C11	0.055 (3)	0.064 (3)	0.071 (3)	−0.006 (3)	0.008 (3)	0.020 (3)
C12	0.062 (3)	0.047 (3)	0.066 (3)	−0.009 (2)	0.011 (2)	0.005 (3)
N	0.045 (2)	0.082 (3)	0.059 (3)	−0.025 (2)	0.0041 (19)	0.000 (2)

### Geometric parameters (Å, °)

**Table:**

Se—O1	1.608 (3)	C6—HC6	0.93
Se—O2	1.622 (3)	C7—C8	1.518 (6)
Se—O4	1.625 (3)	C7—H1C7	0.88 (6)
Se—O3	1.705 (3)	C7—H2C7	1.00 (4)
O3—HO3	0.82	C8—C12	1.381 (6)
C1—C2	1.358 (7)	C8—C9	1.387 (6)
C1—C6	1.369 (7)	C9—C10	1.375 (6)
C1—HC1	0.95 (4)	C9—HC9	0.93
C2—C3	1.371 (7)	C10—N	1.338 (6)
C2—HC2	0.94 (5)	C10—HC10	0.93 (5)
C3—C4	1.391 (6)	C11—N	1.323 (6)
C3—HC3	0.97 (4)	C11—C12	1.358 (7)
C4—C5	1.381 (6)	C11—HC11	1.00 (5)
C4—C7	1.515 (6)	C12—HC12	0.93 (5)
C5—C6	1.382 (6)	N—HN	0.95 (4)
C5—HC5	0.90 (4)		
			
O1—Se—O2	112.56 (15)	C4—C7—C8	112.4 (4)
O1—Se—O4	114.59 (15)	C4—C7—H1C7	109 (4)
O2—Se—O4	111.61 (15)	C8—C7—H1C7	109 (4)
O1—Se—O3	108.78 (16)	C4—C7—H2C7	109 (2)
O2—Se—O3	106.51 (16)	C8—C7—H2C7	107 (2)
O4—Se—O3	101.89 (16)	H1C7—C7—H2C7	110 (5)
Se—O3—HO3	109.5	C12—C8—C9	117.8 (4)
C2—C1—C6	120.1 (5)	C12—C8—C7	120.6 (4)
C2—C1—HC1	118 (3)	C9—C8—C7	121.5 (5)
C6—C1—HC1	122 (3)	C10—C9—C8	119.6 (4)
C1—C2—C3	120.6 (5)	C10—C9—HC9	120.2
C1—C2—HC2	119 (3)	C8—C9—HC9	120.2
C3—C2—HC2	121 (3)	N—C10—C9	119.5 (4)
C2—C3—C4	120.6 (5)	N—C10—HC10	117 (3)
C2—C3—HC3	126 (3)	C9—C10—HC10	123 (3)
C4—C3—HC3	113 (3)	N—C11—C12	119.3 (5)
C5—C4—C3	118.0 (4)	N—C11—HC11	117 (3)
C5—C4—C7	121.0 (4)	C12—C11—HC11	124 (3)
C3—C4—C7	121.0 (5)	C11—C12—C8	121.1 (5)
C4—C5—C6	120.8 (4)	C11—C12—HC12	123 (3)
C4—C5—HC5	119 (3)	C8—C12—HC12	116 (3)
C6—C5—HC5	121 (3)	C11—N—C10	122.6 (5)
C1—C6—C5	119.8 (5)	C11—N—HN	118 (3)
C1—C6—HC6	120.1	C10—N—HN	119 (3)
C5—C6—HC6	120.1		
			
C6—C1—C2—C3	−0.2 (8)	C4—C7—C8—C12	85.0 (6)
C1—C2—C3—C4	2.0 (8)	C4—C7—C8—C9	−93.6 (6)
C2—C3—C4—C5	−2.6 (7)	C12—C8—C9—C10	−0.1 (6)
C2—C3—C4—C7	177.9 (4)	C7—C8—C9—C10	178.5 (4)
C3—C4—C5—C6	1.6 (7)	C8—C9—C10—N	−1.1 (6)
C7—C4—C5—C6	−178.9 (4)	N—C11—C12—C8	−0.2 (7)
C2—C1—C6—C5	−0.8 (8)	C9—C8—C12—C11	0.8 (6)
C4—C5—C6—C1	0.0 (7)	C7—C8—C12—C11	−177.8 (4)
C5—C4—C7—C8	−78.7 (6)	C12—C11—N—C10	−1.1 (7)
C3—C4—C7—C8	100.8 (6)	C9—C10—N—C11	1.8 (7)

### Hydrogen-bond geometry (Å, °)

**Table:**

*D*—H···*A*	*D*—H	H···*A*	*D*···*A*	*D*—H···*A*
O3—HO3···O4^i^	0.82	1.89	2.617 (4)	148
N—HN···O2^ii^	0.95 (4)	1.82 (5)	2.762 (5)	168 (4)

Symmetry codes: (i) *x*, −*y*+3/2, *z*−1/2; (ii) *x*, −*y*+3/2, *z*+1/2.
